# Venous Air Embolism Activates Complement C3 Without Corresponding C5 Activation and Trigger Thromboinflammation in Pigs

**DOI:** 10.3389/fimmu.2022.839632

**Published:** 2022-03-15

**Authors:** Benjamin S. Storm, Judith K. Ludviksen, Dorte Christiansen, Hilde Fure, Kristin Pettersen, Anne Landsem, Bent Aksel Nilsen, Knut Dybwik, Tonje Braaten, Erik W. Nielsen, Tom E. Mollnes

**Affiliations:** ^1^ Department of Anesthesia and Intensive Care Medicine, Nordland Hospital, Bodø, Norway; ^2^ Department of Clinical Medicine, Faculty of Health Sciences, UiT The Arctic University of Norway, Tromsø, Norway; ^3^ Faculty of Nursing and Health Sciences, Nord University, Bodø, Norway; ^4^ Research Laboratory, Nordland Hospital Trust, Bodø, Norway; ^5^ Department of Community Medicine, Faculty of Health Sciences, UiT The Arctic University of Norway, Tromsø, Norway; ^6^ Faculty of Medicine, Institute of Clinical Medicine, University of Oslo, Oslo, Norway; ^7^ Faculty of Health Sciences, KG. Jebsen TREC, UiT The Arctic University of Norway, Tromsø, Norway; ^8^ Department of Immunology, Oslo University Hospital, The University of Oslo, Oslo, Norway; ^9^ Centre of Molecular Inflammation Research, Norwegian University of Science and Technology, Trondheim, Norway

**Keywords:** air embolism, porcine model, complement, inflammation, cytokines, coagulation, thromboinflammation

## Abstract

**Introduction:**

Air embolism may complicate invasive medical procedures. Bubbles trigger complement C3-mediated cytokine release, coagulation, and platelet activation *in vitro* in human whole blood. Since these findings have not been verified *in vivo*, we aimed to examine the effects of air embolism in pigs on thromboinflammation.

**Methods:**

Forty-five landrace pigs, average 17 kg (range 8.5-30), underwent intravenous air infusion for 300 or 360 minutes (n=29) or served as sham (n=14). Fourteen pigs were excluded due to e.g. infections or persistent foramen ovale. Blood was analyzed for white blood cells (WBC), complement activation (C3a and terminal C5b-9 complement complex [TCC]), cytokines, and hemostatic parameters including thrombin-antithrombin (TAT) using immunoassays and rotational thromboelastometry (ROTEM). Lung tissue was analyzed for complement and cytokines using qPCR and immunoassays. Results are presented as medians with interquartile range.

**Results:**

In 24 pigs receiving air infusion, WBC increased from 17×10^9^/L (10-24) to 28 (16-42) (p<0.001). C3a increased from 21 ng/mL (15-46) to 67 (39-84) (p<0.001), whereas TCC increased only modestly (p=0.02). TAT increased from 35 µg/mL (28-42) to 51 (38-89) (p=0.002). ROTEM changed during first 120 minutes: Clotting time decreased from 613 seconds (531-677) to 538 (399-620) (p=0.006), clot formation time decreased from 161 seconds (122-195) to 124 (83-162) (p=0.02) and α-angle increased from 62 degrees (57-68) to 68 (62-74) (p=0.02). In lungs from pigs receiving air compared to sham animals, C3a was 34 ng/mL (14-50) versus 4.1 (2.4-5.7) (p<0.001), whereas TCC was 0.3 CAU/mL (0.2-0.3) versus 0.2 (0.1-0.2) (p=0.02). Lung cytokines in pigs receiving air compared to sham animals were: IL-1β 302 pg/mL (190-437) versus 107 (66-120), IL-6 644 pg/mL (358-1094) versus 25 (23-30), IL-8 203 pg/mL (81-377) versus 21 (20-35), and TNF 113 pg/mL (96-147) versus 16 (13-22) (all p<0.001). Cytokine mRNA in lung tissue from pigs receiving air compared to sham animals increased 12-fold for IL-1β, 121-fold for IL-6, and 17-fold for IL-8 (all p<0.001).

**Conclusion:**

Venous air embolism in pigs activated C3 without a corresponding C5 activation and triggered thromboinflammation, consistent with a C3-dependent mechanism. C3-inhibition might represent a therapeutic approach to attenuate this response.

## Introduction

Venous air embolism, a condition where air inadvertently enters the bloodstream, may complicate many medical and surgical interventions (1–3), or bubbles may form in the bloodstream during rapid decompression when diving (4). Animal studies and human case reports have shown that air embolism triggers acute pulmonary hypertension and hamper pulmonary gas exchange (5–7). Air may also transverse the pulmonary circulation, enter the arterial circulation, disrupt blood circulation, and cause organ ischemia and damage, such as acute myocardial infarction or stroke (6, 8, 9). Patient case reports have described prolonged severe inflammation after air embolism (6, 10), and one case report described a suspected link between perioperative air embolism and disseminated intravascular coagulation, albeit with an unknown pathophysiological mechanism (11). *In vitro* experiments in human serum and heparinized blood have shown that air activates C3 to C3(H_2_0), also termed iC3 (12–14), and *in vitro* studies in lepirudin anticoagulated human whole blood have shown that air emboli trigger a complement C3-driven thromboinflammation (15), which is attenuated by C3 inhibition (15). *Ex vivo* rat studies have shown how air emboli trigger a pulmonary inflammation involving both monocytes and granulocytes and complement (16), recent *in vivo* studies of divers suffering from decompression sickness have shown how microbubbles trigger an acute inflammation (17), and human *in vivo* and *in vitro* studies of bubble-oxygenators has shown that bubbles activate C3 in fully heparinized blood (12). Heparin anticoagulation, however, precludes detailed studies into thromboinflammation and is known to interact with complement in a dosage-dependent manner (18, 19). Despite the numerous *in vitro*, *ex vivo*, and *in vivo* studies on air emboli, the role of the complement system in the air-induced thromboinflammation has to our knowledge, not previously been examined in detail *in vivo* in minimally anticoagulated larger animals.

Measures for the prevention, detection, and immediate treatment of air embolism are well described (3). However, these measures only address the immediate emergency treatment and do not address the management of air-induced thromboinflammation. Based on our recent *in vitro* human whole blood study (15), we hypothesize that C3 activation plays a key role and that C3 inhibition can attenuate the detrimental thromboinflammatory effects of air embolism. Unfortunately, no C3 inhibitor shown to work in swine is available for therapeutic use in porcine studies. Thus, this hypothesis remains untested.

The aim of this exploratory study was to examine the effects of venous air embolism on the complement system, the cytokine network, and coagulation *in vivo* in a porcine model.

## Materials and Methods

We developed a novel porcine model of venous air embolism based on previous animal studies by Durant, Oppenheimer, and Vik (5, 20, 21). The protocol was approved by The Norwegian Animal Research Authority (FOTS ID9466) and performed per the Norwegian Laboratory Animal Regulations and EU directive 2010/64/EU.

### Anesthesia, Instrumentation, and Monitoring

We retrieved 45 Norwegian domestic landrace pigs weighing on average 17 kg (range 8.5-30 kg) from two local farms. Before retrieval, the animals were selected to undergo venous air infusion or serve as sham animals. The animals were anesthetized with azaperone 4 mg, ketamine 500 mg, and atropine 0.5 mg intramuscularly. An iv. cannula was placed in an ear vein, and anesthesia was maintained with a continuous infusion of morphine 2 mg/kg/h, midazolam 0.15 mg/kg/h, and pentobarbital 4 mg/kg/h. We endotracheally intubated the pigs with a Portex ID 6 mm endotracheal tube (Smiths Medical International Ltd, Kent, United Kingdom) and ventilated them with a tidal volume of 10-15 mL/kg, a respiratory rate of 20/minute, an inspired oxygen fraction of 21%, and a positive end-expiratory pressure of 0 cmH_2_O using a Datex-Ohmeda Engström Carestation intensive care ventilator (GE Healthcare, Madison, WI).

We placed a pediatric 9T esophageal echo probe (General Electric, Horten, Norway) in the esophagus and connected it to a Vivid 7 pro echo doppler machine (General Electric). Using sterile cut-down technique, we inserted a 4 Fr. 8 cm Leadercath arterial catheter (Vygon Ltd., Swindon, UK) in the right carotid artery, an 8 Fr. Avanti+ Vascular Sheath Introducer (Cordis, Santa Clara, CA), and a 7.5 Fr. Swan-Ganz CCOmbo pulmonary artery catheter (Edwards Lifesciences Corporation, Irvine, CA) in the right external jugular vein, a 4 Fr. 8 cm PiCCO thermodilution catheter (Pulsion/Getinge, Gothenburg, Sweden) in the femoral artery, and a suprapubic catheter with a temperature sensor in the bladder. The pulmonary artery catheter, sheath introducer, and arterial cannula were connected to a TruWave x3 T001660A Pressure Monitoring Set (Edwards Lifesciences Corporation), and the PiCCO catheter was connected to a PV8215 PiCCO Monitoring Kit (Pulsion). Both systems were connected to a pressurized 500 mL Ringer’s acetate bag (Fresenius Kabi, Oslo, Norway) with 1250 IU Heparin (LEO Pharma AS, Oslo, Norway) added to a final concentration of 2.5 IU/mL. A continuous infusion of 3 mL/h of heparinized Ringer’s acetate was delivered through each of the four pressure lines, i.e., 30 IU Heparin pr hour was administered to the animals. If flushing of the catheters was needed, normal saline was used. The catheters were not flushed with heparinized Ringer. After instrumentation, the animals were stabilized for thirty minutes before starting the air infusion or sham observation.

We recorded continuous invasive arterial, pulmonary, and venous blood pressures, ECG, plethysmographic arterial saturation (SpO_2_), end-tidal expired CO_2_ (EtCO_2_), and hourly intermittent cardiac output by thermodilution using an Intellivue MP70 monitor (Philips Healthcare, Cambridge, CA) as per manufacture’s instructions. Additionally, hourly intermittent and continuous cardiac output was measured using the Pulsion Pulsed index Continuous Cardiac Output PiCCO2 monitor (Pulsion) as per manufactures instructions. Before the experiments, we performed echocardiography, and throughout the experiments, cardiac function and buildup of air and thrombi in the pulmonary artery and systemic egress of air were monitored using continuous echocardiography.

### Exclusion Criteria

**Figure 1:**
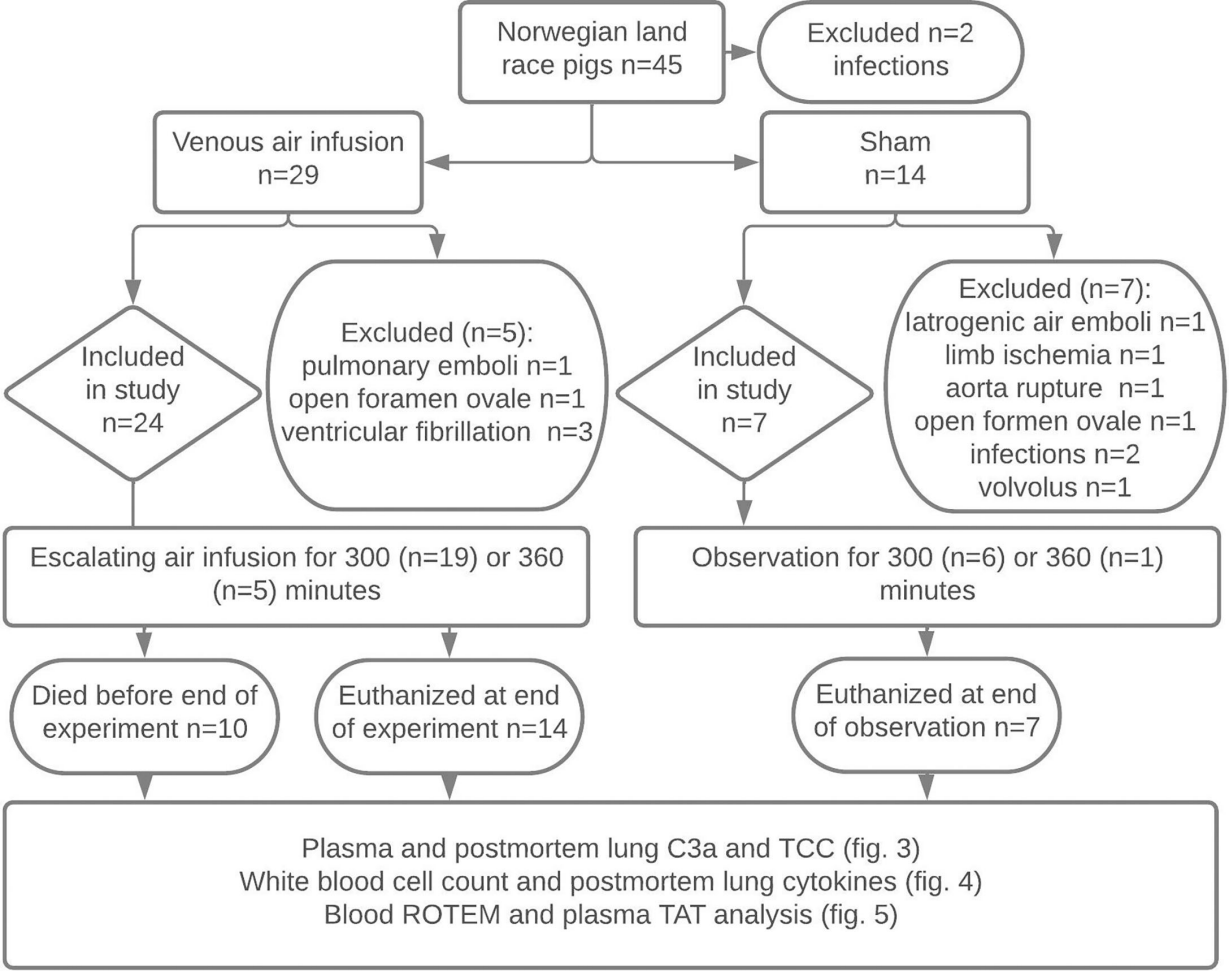
Experimental flowchart. Forty-five Norwegian landrace pigs from two farms were selected by convenience sampling to undergo 300 or 360 minutes of titrated air infusion or serve as sham animals. Animals with infections, pulmonary emboli during animal preparation, persistent foramen ovale, ventricular fibrillation, iatrogenic venous air embolism, limb ischemia, or iatrogenic aortic rupture were excluded. Ten pigs receiving air infusion died due to the air infusion after a median infusion time of 243 minutes. Animals alive at the end of the experiments were euthanized. Blood was sampled at regular intervals throughout the experiments, and lung tissue was sampled post-mortem. Blood and lung samples were analyzed as detailed in the figure.

Animals with infections, either observed upon retrieval or diagnosed at the post-mortem lung autopsy or defined as a white blood cell count or complement C3a level three standard derivations above mean baseline values, were excluded (**Figure 1**). Animals with persistent foramen ovale, detected by preoperative echocardiography with an agitated saline injection or by post-mortem autopsy, and animals with iatrogenic complications, such as ventricular fibrillation upon inflation of the pulmonary artery catheter or vessel injury due to cannulation, were excluded from the study. Also, one animal in the sham group was excluded due to severe volvulus, which became evident during the observation period, and one due to an unintended infusion of air through an unpurged intravenous line.

### Air Infusion

**Figure 2:**
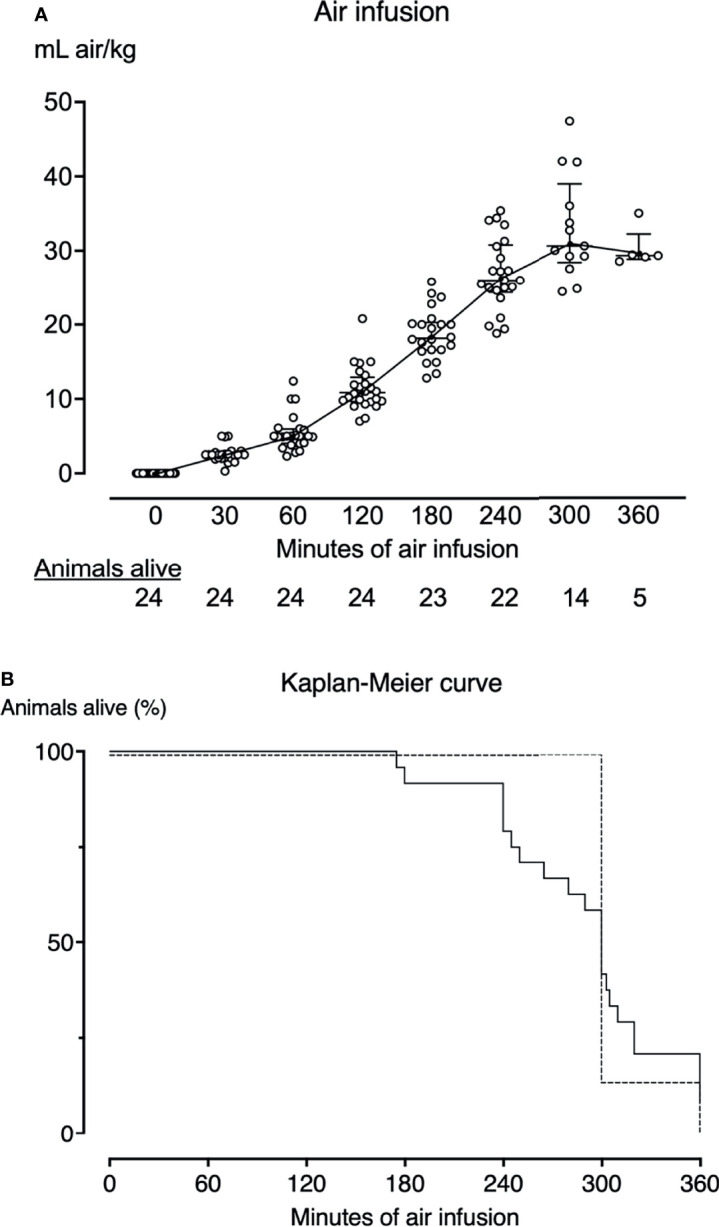
Air infusion protocol and survival curves. **(A)** Twenty-four pigs received intravenous air infusion based on body weight. The infusion rate was increased hourly. Each point represents one pig. The horizontal lines indicate the median infusion rate. Error bars span the interquartile range. Animals are only included in the graph until death. Pigs alive at each sample point are listed below the graph. **(B)** Kaplan-Meier survival curve of the animals that received air infusion (solid line) and sham animals (dotted line). Pigs that received air infusions died after a median of 300 minutes (IQR 249-320). All sham animals lived until the end of the observation period of either 300 (n=6) or 360 minutes (n=1).

After instrumentation and baseline blood sampling, we administered a continuous air infusion of 3-7 mL/kg/hour by syringe driver through an ear vein. The infusion rate was either maintained or increased with 1-2 mL/kg every sixty minutes for 300 minutes (21 pigs) or 360 minutes (3 pigs) or until the animals died (**Figure 2A**). The air infusion protocol was based on previous studies (5, 20, 21) and titrated to cause severe hemodynamic instability, but not systemic egress of air embolism or the death of the animals. Initially, a 360-minute observation time was chosen to allow adequate time for the synthesis of cytokines. To reduce the preterm mortality of animals, this was reduced to 300 minutes after the initial experiments. We adjusted the respiratory tidal volume, respiratory rate, and inspired oxygen fraction throughout the experiments to maintain arterial pH 7.34-7.40 and SpO_2_ above 90%. We infused 2-3 mL/kg/h of Ringer’s acetate to compensate for insensible fluid losses. If mean arterial pressure dropped below 55 mmHg, we administered repeated boluses of 100 mL Ringer’s acetate and infused noradrenaline (Abcur, Helsingborg, Sweden) in the range 0.01 to 0.8 μg/kg/minute. Animals still alive at the end of the experiment were euthanized by intravenous injection of 30-50 mmol potassium chloride (B. Braun, Melsungen, Germany).

### Blood and Tissue Sampling and Analysis

We sampled arterial blood from the carotid artery at baseline (just before the air infusion was started at zero minutes), after 30, 60, 180, 240, and 300 minutes of air infusion, and at the end of the experiments. Approximately 100 mL of blood was sampled from the animals throughout the experiments. The blood was sampled using a Vacutainer closed vacuum system, Vacuette EDTA tubes and 3.2% sodium-citrate tubes, and serum tubes with gel (all Vacuette, Greiner Bio-One GmbH Frickenhausen, Germany), and a safePICO heparinized blood gas syringe (Radiometer, Copenhagen, Denmark). To avoid unintended contamination of the samples with heparin from the line flush solution, 5 mL of blood was aspirated into a sterile syringe immediately before sampling. Additionally, after drawing blood into the heparinized blood gas syringe, 2 mL of blood was drawn and discarded before subsequent sampling. Blood drawn on heparinized syringes was analyzed immediately after drawing for lactate, pH, pO_2,_ and pCO_2_ on the ABL80 Flex blood gas analyzer (Radiometer, Copenhagen, Denmark). A set of EDTA tubes were stored at room temperature for up to 8 hours and analyzed for white blood count using the ADVIA 2120i (Siemens Healthcare GmbH, Erlangen, Germany) or IDEXX ProCyte Dx (IDEXX Laboratories, Westbrook, ME). A set of EDTA tubes were immediately centrifuged at 4°C at 1500 g for 15 min and plasma isolated and frozen at -80°C for later analysis of complement and cytokines, as detailed below. PAXgene tubes were carefully tilted ten times, left at room temperature for a minimum of two hours, frozen at -20°C overnight, and then at -80°C until RNA extraction and cytokine mRNA analysis.

Post-mortem, we opened the thorax and sampled tissue from the lower lobe of the left lung. These samples were snap-frozen on dry ice in NUNC tubes (Thermo Scientific, Roskilde, Denmark) with no additive for further homogenization and analysis.

### Homogenization of Lung Tissue

For cytokine mRNA analysis, approximately 20 mg of tissue was transferred to gentleMACS M-tubes (Miltenyi Biotec, Bergisch Gladbach, Germany), and 800 µL Trizol reagent (Thermo Fisher Scientific, MA) was added to the samples. The samples were homogenized using program 7 on the Dispomix homogenizer (Miltenyi Biotec). After homogenization, the samples were left at RT for 5 minutes, centrifuged at 1,400 *g* for two seconds, and transferred to 1.5 mL Eppendorf PCR Clean Safe-Lock Tubes (Eppendorf, Enfield, CT) and stored at -80°C for later mRNA isolation and PCR analysis.

For complement analysis, approximately 100 mg of tissue was transferred to gentleMACS M-tubes, and a mixture of 10 µL Protease Inhibitor Cocktail Set I (Merck KGAA, Darmstadt, Germany) and 1 mL CytoBuster Protein Extraction Reagent (Millipore Sigma, Burlington, MA) was added to the samples, and the samples were homogenized using program 7 on the Dispomix homogenizer (Miltenyi Biotec). After homogenization, the samples were incubated for 5 minutes on ice, centrifuged for 20 minutes at 2,500 *g* at 4°C, and the supernatant was transferred to 1 mL Matrix tubes (Thermo Fisher Scientific) and stored at -80°C for later analysis. Likely, receptor-bound C3a detached during the lysis process, making it available for subsequent ELISA detection.

### Analysis of Complement in Plasma and Lung Tissue

We measured complement C3a using ELISA with porcine-specific C3a monoclonal antibodies as previously described in detail (22). The antibody binds to a neoepitope exposed when C3a is cleaved off C3, and the assay only detects free C3a in the fluid phase. We measured TCC using ELISA with the anti-human-C9 neoepitope antibody clone aE11 produced in-house as capture antibody and a porcine cross-reacting anti-human C6, Quidel (San Diego, CA) as detection antibody as described in detail (23, 24). We have previously documented that the aE11 cross-reacts with porcine TCC (23).

### Analysis of Cytokines in Plasma

We analyzed EDTA plasma for the following cytokines using immunoassays: Tumor necrosis factor (TNF) and interleukin (IL)-6 using the Porcine TNF and IL-6 Quantikine sandwich ELISA kit (R&D Systems Inc.) with optical density measured by Infinite M200 Pro microplate reader (Tecan Trading AG, Switzerland); IL-1β, IL-6, and IL-8 using a porcine MILLIPLEX map Kit (Merck, EMD Millipore Corporation, Billerica, MA) and IL-10 using Invitrogen ProcartaPlex Multiplex Porcine Immunoassay (Bender MedSystems GmbH, Vienna, Austria), and the fluorescence intensity analyzed on a Bio-Plex 200 Multiplex Analyzer (Bio-Rad Laboratories). All analyses were performed in accordance with the manufacturer’s instructions.

### Analysis of Cytokines in Lung Tissue

We analyzed samples of homogenized lung tissue for IL-6 using the Milliplex map Kit (Merck) and IL-10 using Invitrogen ProcartaPlex Multiplex Immunoassay for Porcine assay (Bender MedSystems GmbH, Vienna, Austria). The fluorescence intensity was analyzed on a Bio-Plex 200 Multiplex Analyzer (Bio-Rad Laboratories). We analyzed the homogenized tissue for TNF, IL-1β, and IL-8 using Quantikine sandwich ELISA kits (R&D Systems Inc.) and measured the optical density using an Infinite M200 Pro microplate reader (Tecan Trading AG). All analyses were performed in accordance with the manufacturer’s instructions. Results are given pr. mL homogenate.

### Analysis of Cytokine mRNA in Lung Tissue

Paxgene blood, 1.5 mL, was transferred to 2 mL Eppendorf tubes (Eppendorf) and centrifuged at 5,000 *g*, 10 min. The supernatant was carefully poured from each tube, and the pellet was resuspended in 1 mL nuclease-free water. The RNA was re-pelleted by centrifugation at 5,000 *g* for 10 minutes. MagMAX for Stabilized Blood Tubes RNA Isolation Kit (Thermo Fisher Scientific) was used for total RNA isolation in accordance with the manufacturer’s instructions. The extracted RNA was eluted in 50 µL of Elution Buffer, quantified using Thermo Scientific Nanodrop 2000 (Thermo Fisher Scientific), and controlled with the Agilent 2100 Bioanalyzer (Agilent Technologies Santa Clara, CA). The mean RNA Integrity Number was 9.6.

RNA was extracted from homogenized lung tissue using TRIzol Reagent and RNeasy MinElute Cleanup kit (Qiagen, Hilden, Germany) and subsequent DNase treatment (Thermo Fisher Scientific) as described previously (25). RNA was quantified using the NanoDrop 2000 and integrity controlled using the Agilent 2100 BioAnalyzer. The mean RNA integrity number was 8.1.

For gene expression studies, the TaqMan RNA-to-Ct 1-step Kit (Thermo Fisher Scientific) was used. The amount of input RNA was 25 ng in a total volume of 20 µL. Cycling conditions were set according to the kit insert, and qPCR was run in triplicates for each candidate gene in MicroAmp Fast 96-Well Reaction Plates (all reagents from Thermo Fisher Scientific) using QuantStudio 6 Flex Real-Time PCR System instrument (Thermo Fischer Scientific). Predeveloped TaqMan porcine gene expression assays (Thermo Fisher Scientific) for the following candidate genes were used: IL-1β (Ss03393804_m1), IL-8 (Ss03392437_m1), and TNF (Ss03391318_g1). IL-6 (PIG_IL6) was custom-made by Thermo Fisher Scientific. Ribosomal Protein S 18 (Ss03391029_g1) was used as endogenous control and was stably expressed in all samples. The relative quantification (RQ) of cytokine mRNA expression was calculated using the comparative cycle threshold (2^-ΔΔCt^) method. All results are presented as fold change (RQ) compared to sham animals.

### Analysis of Thrombin-Antithrombin Complex (TAT) in Plasma

We quantified the TAT in EDTA plasma using the human Enzygnost TAT micro (Siemens Healthcare Diagnostics Products GmbH, Marburg, Germany), documented to cross-react with porcine TAT (26) in accordance with the manufacturer’s instructions. The optical density was measured using an Infinite M200 Pro microplate reader (Tecan Trading AG).

### Whole Blood Rotational Thromboelastometry (ROTEM)

We used the ROTEM delta (Tem Innovations GmbH, Munich, Germany) to analyze the kinetic of the clot formation. Citrated blood sampled at 0, 30, 60, and 120 minutes of air infusion were incubated at 37°C for five minutes and analyzed using the non-activated thromboelastometry test (NATEM) according to the manufacturer’s instructions. 300 µL citrated blood was added in a disposable cup with 20 µL star-TEM reagents containing CaCl_2_ (Tem Innovations GmbH). The clot formation was detected by inhibition of the movement of the pin in the cup, and several parameters were measured or calculated. Clotting time (CT, in seconds), clot formation time (CFT, in seconds), α-angle (in degrees), and maximum clot formation (MCF, in millimeters) were analyzed in this study.

### Data Analysis and Statistical Methods

All data was collected and organized, and missing values were imputed in Microsoft Excel for Mac version 16.54 (Microsoft Inc., Redmond, CA). In animals that died before 300 minutes of observation, missing data were imputed using the Last-Observation-Carried-Forward method. Changes from baseline were calculated for leukocytes, TAT, and ROTEM readouts, and RQ were calculated for cytokine mRNA qPCR in Microsoft Excel. Statistical analysis and data charting was done in Prism for macOS version 9.3.0 (Graphpad Software, La Jolla, CA). Results from post-mortem analysis of lung cytokines, cytokine mRNA, and complement in pigs receiving air infusion were compared with sham animals using the two-tailed Mann-Whitney U-test. Changes from baseline to time of death in plasma complement, TAT, and leukocytes and changes from baseline to 120 minutes of observation in whole blood ROTEM were analyzed using the Wilcoxon matched-pairs signed-rank test. All analyses were corrected for multiple comparisons using Benjamini and Hochberg’s original false discovery rate (FDR) method with an FDR of 1%. A p-value <0.05 was considered significant. Results are presented as medians with interquartile range. Graphs of repeated samples are presented as changes from baseline. As our data did not follow a Gaussian, data are presented as medians with interquartile range. qPCR relative quantifications are presented as geometric means with 95% CI.

Five pigs had one or more plasma TCC results below the lower detection limit, and one pig had lung tissue TCC results below the lower detection limit. These missing values were imputed with a random number between 0.01 and the lower detection limit. Six pigs had lung cytokines results below the lower detection limit but with reliable results on the extrapolated standard curve. These results were included in the analysis. One lung tissue IL-8 analysis failed and yielded no result. This missing value was replaced with the median lung tissue IL-8 from all pigs. One pig had an erroneous ROTEM baseline reading due to a heparin bolus given shortly before the baseline sampling. The baseline ROTEM for this animal was imputed by averaging the ROTEM readings thirty minutes before and thirty minutes after baseline sampling.

## Results

### Animals Included in the Study

Fourteen of 45 animals were excluded from the study due to infections, open foramen ovale, or perioperative adverse events (**Figure 1**). Twenty-four animals receiving air infusion and seven sham animals were included in the study. Baseline observations sampled after the initial instrumentation but before air infusion did not differ between pigs allocated to air infusion and sham animals (**Table 1**).

**Table 1:** Characteristics of pigs included in the study at baseline^1^.

		Pigs receiving air infusion (n=24) Median (IQR)	Sham animals (n=7) Median (IQR)	p^2^
Weight	kg	17 (10-24)	11 (9.5-22)	0.8
*ROTEM*				
Clotting time (CT)	seconds	613 (531-677)	519 (453-604)	0.2
Clot formation time (CFT)	seconds	161 (122-195)	133 (126-167)	0.6
α-angle	degrees	57 (62-68)	66 (59-68)	0.6
Maximum clot formation (MCF)	millimeter	64 (59-73)	65 (63-68)	0.6
*Biochemistry*				
White blood cell count (WBC)	x10/^9^L	17 (10-24)	16 (11-18)	0.6
*ELISA*				
Thrombin-antithrombin complex (TAT)	µg/mL	35 (28-42)	23 (16-29)	0.2
C3a	ng/mL	21 (15-46)	12 (7.9-20)	0.2
TCC	CAU/mL	0.8 (0.6-1.1)	0.7 (0.3-0.8)	0.4

^1^Sampled after 30 minutes after instrumentation, before air infusion (T0). ^2^Wilcoxon signed-rank test. p values corrected for multiple comparisons using Benjamin and Hochberg’s FDR method.

Despite careful titration of the air infusion, ten of the 24 animals (42%) receiving air infusion died before the end of the experiments with a clinical picture of acute right heart failure, after a median of 243 minutes (IQR 240-261) (**Figure 2B**). Fourteen of the 24 animals receiving air infusion, and all sham animals lived until the end of the experiments. The last observations were carried forward for the ten animals that died before 300 minutes of observation.

### Complement in Plasma and Lung Tissue

**Figure 3:**
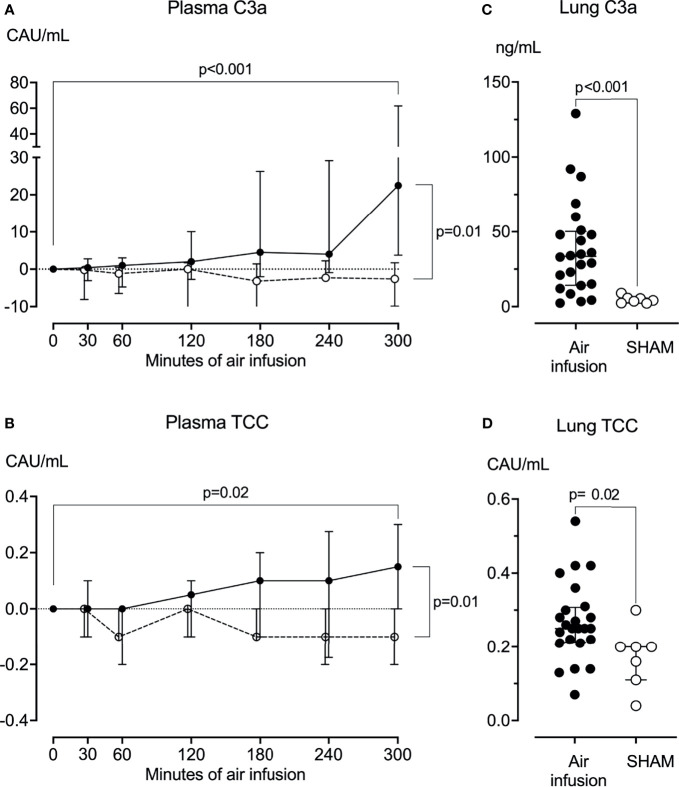
Complement activation products in plasma and lung tissue. **(A)** Plasma C3a was measured at regular intervals throughout the experiments. C3a increased significantly from baseline in animals receiving air infusion (solid line) but not in sham animals (dotted line). **(B)** Plasma TCC measured at regular intervals throughout the experiments increased significantly from baseline in animals receiving air infusion (solid line) compared to the sham animals (dotted line). **(C)** In lung tissue sampled post-mortem, C3a was significantly higher in animals receiving air infusion than in sham animals. **(D)** In lung tissue sampled post-mortem, TCC was slightly but statistically significantly higher in animals receiving air infusion than sham animals. Dots in panels **(A, B)** and horizontal lines in panels **(C, D)** represent medians. Error bars span the interquartile range. Note Y-axis is split in panel **(A)** The two-tailed Mann-Whitney test was used between groups, and the Wilcoxon signed ranks test for within-group comparisons. P-values in panels **(A, B)** are corrected for multiple comparisons using Benjamin and Hochberg’s FDR method.

In pigs receiving air infusion, baseline median plasma C3a was 21 ng/mL (IQR 15-46). Plasma C3a increased steadily after 120 minutes of air infusion and was 3.2-fold higher (67 ng/mL [IQR 39-84]) at death than at baseline (p<0.001) (**Figure 3A**). In sham animals, no C3a increase was observed during the observation period. In pigs receiving air infusion, baseline median plasma TCC was 0.8 CAU/mL (IQR 0.6-1.1). During the experiment, plasma TCC increased to a lesser extent and was 1.25-fold higher (1.0 CAU/mL [IQR 0.7-1.4]) at death than at baseline (p=0.02) (**Figure 3B**). In sham animals, no TCC increase was observed during the observation period.

In post-mortem lung tissue samples from pigs that received air infusion, median C3a was 34 ng/mL (IQR 14-50) versus 4.1 ng/mL (IQR 2.4-5.7) in sham animals (p<0.001) (**Figure 3C**), and median TCC was 0.3 CAU/mL (IQR 0.2-0.3) in pigs that received air infusion versus 0.2 CAU/mL (IQR 0.1-0.3) in the sham animals (p=0.02) (**Figure 3D**).

### White Blood Cell Count

**Figure 4:**
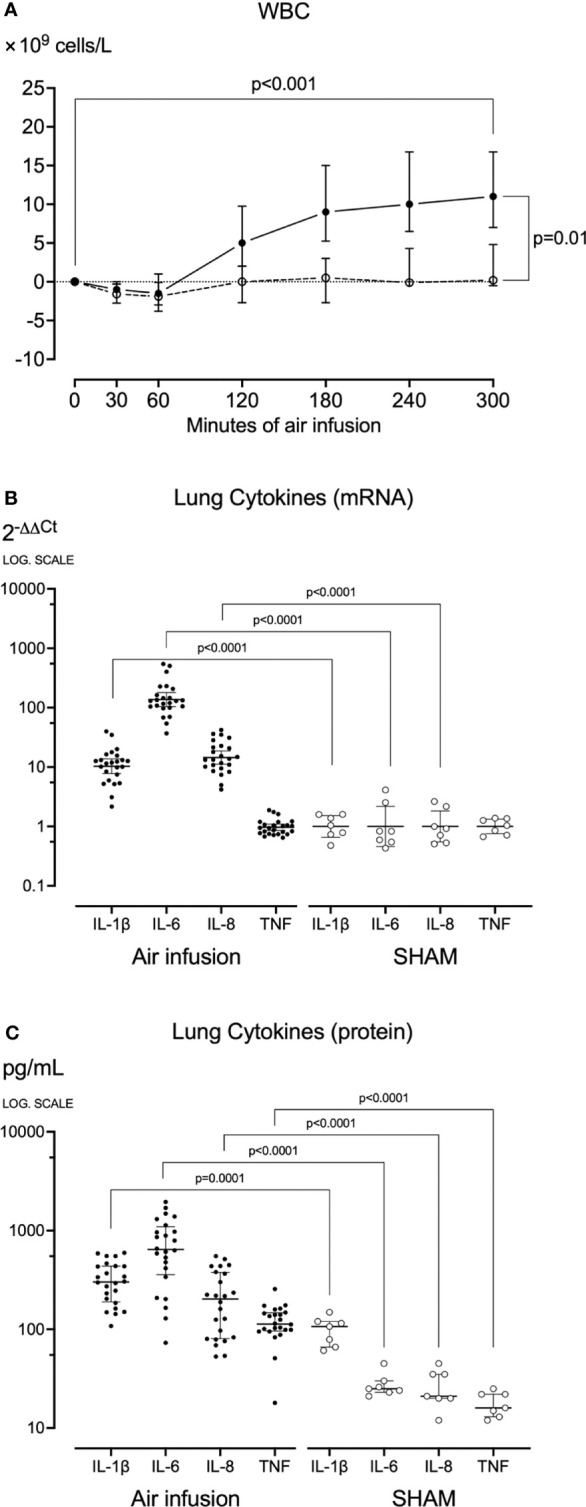
Blood White Cell Count and Lung cytokines. **(A)** White blood cells were counted at regular intervals throughout the experiments. After sixty minutes of air infusion, white blood cells increased significantly from baseline in animals receiving air infusion (solid line) but not in sham animals (dotted line). **(B)** In lung tissue sampled post-mortem cytokine mRNA was analyzed using qPCR, and results were calculated using the 2^-ΔΔCt^ method. **(C)** In lung tissue sampled post-mortem, cytokines were measured using ELISA or multiplex. Animals receiving air infusion are represented as closed circles and solid lines, and sham animals as open circles and dotted lines. Circles in panel **(A)** and horizontal lines in panel **(C)** represent medians, and error bars span the interquartile range. The horizontal lines in panel **(B)** represent the geometric means, and error bars span the 95% CI of the mean.

In pigs receiving air infusion, baseline median plasma white blood cell count was 17·10^9^ cells/L (IQR 10-24). Plasma white blood cell count increased steadily after 120 minutes of air infusion and was 1.6-fold higher (28·10^9^ cells/L [IQR 16-42]) at death than at baseline (p<0.001) (**Figure 4A**). In sham animals, only a slight increase in white blood cell count was noted.

### Cytokines in Plasma and Lung Tissue

During the experiments, no significant changes in plasma cytokines were observed in pigs receiving air infusion or sham animals. In contrast, in pigs receiving air infusion compared to sham animals, the post-mortem mean lung tissue cytokines mRNA expressions were increased: 12-fold for IL-1β, 121-fold for IL-6, and 17-fold for IL-8 (all p<0.001) (**Figure 4B**). The TNF mRNA expression did not differ between the groups. In line with the mRNA findings, the median lung tissue cytokines measured as protein in pigs receiving air infusion compared to sham animals were: IL-1β 302 pg/mL (IQR 190-437) versus 107 pg/mL (IQR 66-120), IL-6 644 pg/mL (IQR 358-1094) versus 25 pg/mL (IQR 23-30), IL-8 203 pg/mL (IQR 81-377) versus 21 pg/mL (IQR 20-35), and TNF 113 pg/mL (IQR 96-147) versus 16 pg/mL (IQR 13-22) (all p<0.001) (**Figure 4C**). Thus, TNF increased as measured by protein but not by mRNA.

### Coagulation

**Figure 5:**
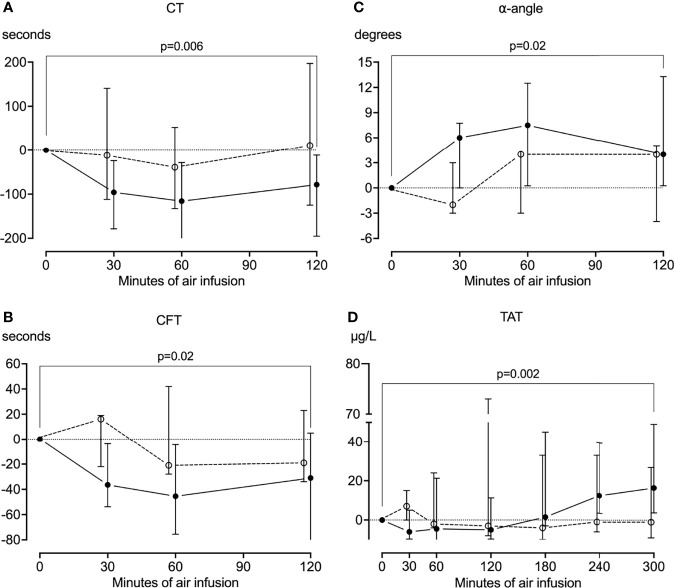
Coagulation. Whole blood was drawn from the animals every half hour for the first two hours of the experiments, and the coagulation was analyzed using rotational thromboelastometry (ROTEM). In animals receiving air infusion, a significant reduction from baseline in the clotting time **(A)** and clot formation time **(B)** and a significant increase from baseline in the α-angle **(C)** was observed. These parameters mainly remained at baseline in sham animals, albeit with a pronounced heterogenicity. **(D)** Plasma was drawn from the animals throughout the experiments and analyzed for thrombin-antithrombin (TAT) using ELISA. TAT increased significantly from baseline in pigs receiving air infusion and remained at baseline in sham animals. Solid line represents pigs receiving air infusion. Dotted lines are sham animals. Circles represent medians, and error bars span the interquartile range.

In pigs that received air infusion, baseline median clotting time was 613 seconds (IQR 531-677) (**Figure 5A**), baseline median clot formation time was 161 seconds (IQR 122-195) (**Figure 5B**), and baseline median α-angle was 62 degrees (IQR 57-68) (**Figure 5C**). Already after 30 minutes of air infusion, clotting time and clot formation time had decreased and α-angle increased, and after 120 minutes of air infusion, both clotting time and clot formation time were significantly reduced compared to baseline, to 538 seconds (IQR 399-620) and 124 seconds (IQR 83-162) (p=0.006 and p=0.02, respectively). The α-angle significantly increased compared to baseline, to 68 degrees (IQR 62-74) (p=0.02). In contrast, in sham animals, neither clotting time, clot formation time, or α-angle changed significantly during the observation period (**Figures 5A–C**). The maximum clot formation did not change significantly during the experiments.

In pigs that received air infusion, baseline median TAT was 35 µg/mL (IQR 28-42). TAT increased after 180 minutes of air infusion and continued to increase throughout the experiments (**Figure 5D**). At the end of the experiments, median TAT was 1.5-fold higher (51 µg/mL [IQR 38-89]) than at baseline (p=0.002). In contrast, TAT remained at baseline in sham animals throughout the observation period.

## Discussion

In this study, we have shown *in vivo* in a porcine model how venous air embolism triggered a thromboinflammation with activation of the complement system, leukocytosis, release of proinflammatory cytokines, and activation of coagulation. Notably, air embolism triggered a relatively selective and robust C3 activation as measured by C3a, without a corresponding C5 activation, as measured by TCC.

The complement system is an integral part of the innate immune system, protecting the body against invading pathogens and endogenous cellular damage. The system consists of several plasma peptides, which bind to antibodies, lectins, or foreign surfaces, resulting in classical, lectin, or alternative pathway activation, respectively (27). It has previously been shown that air activates the complement system mainly through the alternative pathway (14, 15) by hydrolysis of C3 to generate C3(H_2_O), also termed C3b-like or iC3 (14). This initial alternative C3 activation generates the C3(H_2_O)B, where factor B is cleaved by factor D to form Ba, which is released, and Bb, which together with properdin (P) binds to C3(H_2_O) and forms the first alternative C3 convertase, C3(H_2_O)BbP. This convertase further activates C3 to be cleaved to C3b and C3a, and the final alternative pathway C3 convertase C3bBbP is formed. Under normal circumstances, C3bBbP forms the C5-convertase (C3b_2_BbP), which catalyzes the formation of the terminal C5b-9 complement complex (TCC). Activated C3- and C5-split products have been shown to interact with thrombocytes and leukocytes (28, 29) and may potentially trigger a *thromboinflammation* involving both leukocytes, platelets, and coagulation. Interestingly, and in line with our previous *in vitro* findings in human whole blood (15), we discovered that air embolism predominantly activated C3 with only a minor C5 activation, contrary to what is otherwise observed in general when complement is activated, for example, by bacteria or damage or pathogen-associated molecular patterns. The C5a-C5aR axis is known to play an important role in complement-mediated thromboinflammation associated with a wide array of diseases (27, 30), and the monoclonal anti-C5 antibody eculizumab has been used clinically for many years to treat complement-driven diseases, such as paroxysmal nocturnal hemoglobinuria and atypical hemorrhagic uremic syndrome (31). C3a receptors have been shown on both platelets and activated astrocytes, neutrophils, and monocytes (32, 33), and we have previously shown how C3 inhibition attenuates air-induced thromboinflammation (15). Recently, a specific C3 inhibitor, pegcetacoplan (Empaveli, Apellis Pharmaceuticals, Waltham, MA), was approved to treat paroxysmal nocturnal hemoglobinuria (34), and further studies should evaluate if C3 inhibitors can be used to dampen thromboinflammation triggered by air embolism.

The aforementioned observed relative selective C3 activation without a corresponding C5 activation is most likely related to the mechanism by which air activates C3. The efficacy of the complement system convertases depends on whether the C3 activation occurs on the solid phase, where C3 binds to surfaces to generate a potent convertase, or in the fluid phase, where a less potent convertase is formed. As mentioned above, air bubbles have been shown to generate a fluid-phase C3 convertase *in vitro* in plasma experiments (14), and it is reasonable to assume that an identical fluid-phase convertase is formed *in vivo* in pigs with air embolism. The formation of a less efficient fluid-phase convertase could explain why the C5 convertase is insufficiently generated, which we documented with limited TCC formation compared to C3 activation. This contrasts the complement activation otherwise seen. For example, in a porcine study of polymicrobial sepsis (35), both C3a and TCC increased substantially, with C3a appearing first followed by TCC (22).

Another interesting imbalance between C3 and C5 convertase potency occurs when autoantibodies, nephritic factors (NeFs) form against the complement convertases, as recently reviewed (36). NeFs may bind to the C3, C4, or C5 convertase (C3NeF, C4Nef, and C5Nef, respectively). NeF binding stabilizes the convertases, resulting in pathological ongoing complement activation. As with air embolism, we have previously shown that some C3NeFs activate C3 without a corresponding C5 activation (37). NeFs cause an autoimmune kidney failure in patients, particularly if the C5 is activated (36).

The imbalance between C3 and C5 activation is important concerning the potential treatment of patients with air embolism. Most complement-mediated diseases involve the C5a or the C5b-9, and treatment involves inhibition of C5 to keep the C3 open for complement-driven bacterial defense. However, in the case of venous air embolism, C3 inhibition would be required to reduce the thromboinflammatory response effectively. It remains to be shown if other pathophysiological conditions are similarly mainly driven by C3 activation with only a modest activation of C5.

In our study, we measured C3a using an ELISA assay with a porcine-specific antibody (22) and TCC using a human assay shown to work well in swine (23, 24), as no reliable porcine C5a assay is commercially available. This is a reasonable approach, as C5a and TCC (sC5b-9) are released in equimolar concentration in plasma. TCC has a plasma half-life of 50-60 minutes (38) compared to C5a with a half-life of one minute (23), making it a robust marker of terminal pathway activation. Further details on assays for the detection of complement activation products in humans and animals are reviewed in detail (39).

In pigs that received air infusion, but not in sham animals, we found increased levels of proinflammatory cytokines in lung tissue, increased circulating white blood cells, and coagulation system activation. Combined with our human *in vitro* findings (15), we have shed new light on the mechanisms for inflammation, coagulation, and the interplay between complement, inflammation, and coagulation. Our data suggest that complement activation played an important role mainly through the activation of C3. *In vitro*, we showed how selective inhibition of C3 reduced complement activation, cytokine release, and coagulation. Most of the inflammatory mediators were purely mediated by C3-activation, but a few were C5a dependent. The majority of mediators were abolished by C3 inhibition. Thus, there is a theoretical rationale for C3 inhibition as a treatment of air embolism *in vivo*. Unfortunately, as no inhibitor reacting with porcine C3 is currently available largescale for *in vivo* studies, we could not verify these findings in our porcine model.

The accumulation of C3a and increased cytokine concentrations in lung tissue of pigs with air embolism indicates the importance of the lungs in the pathophysiology of venous air embolism and air-induced thromboinflammation. Venous air emboli are transported with the bloodstream to the lung capillaries, where the air is absorbed into the alveoli and exhaled. However, large amounts of air may overwhelm the lung’s filtering capacity, occlude the capillaries and obstruct blood flow, trigger a local inflammatory process, or transverse the lungs to the systemic circulation (7, 9, 40). In humans, air bubbles have been shown to activate the alternative complement pathway *in vivo, ex vivo*, and *in vitro* (12, 14, 15). We suggest that lodged air bubbles in the pig’s lung capillaries activated C3 by a similar mechanism and subsequently triggered inflammation and coagulation alike in humans. This would explain the increase in lung tissue cytokines and circulating white blood cells observed in pigs receiving air infusion. The pulmonary inflammation may have triggered lung edema. The combination of air bubbles, thrombi, and edema may have further hampered blood flow and gas exchange, resulting in pulmonary hypertension, a drop in end-tidal CO_2_, and acute right heart failure, as we and others have found in animals studies and human cases (6, 20, 41, 42).

In our study, air infusion triggered coagulation, measured by ROTEM and TAT. We recently showed *in vitro* in human whole blood that coagulation was activated through a complement-dependent mechanism, where C3 activation played a pivotal role, and through a complement-independent mechanism, which we did not identify in detail (15). We speculate that air embolism activated the coagulation through similar mechanisms *in vivo* in pigs. The ROTEM and TAT results were most likely diminished and underestimated, as we had to administer a heparin infusion of approximately 30 IU/h to avoid coagulation of arterial and venous lines and the buildup of thrombi on the pulmonary artery catheter. Additionally, in animals receiving air infusion, but not in sham animals, we also had to administer intermittent heparin boluses of approximately ten IU due to thrombi in the pulmonary artery catheter.

Despite many similarities between humans and pigs, human immunoassays may not reliably cross-react and work in pigs. In this study, we limited complement readouts to C3a and TCC, and we used only cytokine assays shown to work in pigs as detailed in (43). Many commercial immunological assays, including complement and cytokine assays, are marketed as working in pigs. Over the years, we have thoroughly tested most available complement assays, comparing them to the reliable C3a and TCC assays described above and in (22–24); disappointingly, in such comparisons, most assays failed to detect complement activation reliably. Likewise, we have recently studied porcine-specific cytokine assays, finding that many of these are also unreliable (43).

Our study has some limitations; it was a single-center, non-randomized, and non-blinded exploratory study. The animals were assigned to air infusion or to serve as sham animals at the examiner’s discretion. However, we do not suggest that these limitations affected our results.

Despite careful titration of the air infusion, several animals died before the intended 300 minutes of air infusion, with most premature deaths occurring between 240 and 300 minutes of air infusion (**Figure 2**). These premature deaths could have been mitigated by using a less-aggressive air infusion protocol, for example, by not increasing the infusion rate after 180 minutes of air infusion or by terminating the experiments after 180 minutes of air infusion. However, we titrated the air infusion to maximize the effects of air embolism on the thromboinflammation to allow for complement production and time for the synthesis of cytokines. It is possible that reducing the air infusion or shortening the experiments would have reduced complement readouts and yielded lower post-mortem cytokine levels. However, we measured cytokines by mRNA and by protein quantification, enabling us to detect cytokines with high sensitivity. Cytokine mRNA forms rapidly before proteins are synthesized, but, as we observed concerning TNF and IL-1β, may also be downregulated and thus not detected by PCR when proteins are still present and detected by ELISA in the tissue. In our study, pigs that died prematurely were more inflammatory affected than animals surviving the experiment, and it is unlikely that premature death impacted our results negatively.

## Conclusion

Venous air embolism triggered thromboinflammation *in vivo* in pigs, reflected by increased plasma complement C3 activation, leukocytosis, and coagulation. Furthermore, post-mortem pulmonary tissue homogenates revealed increased C3a and cytokines IL-1β, IL-6, IL-10, and TNF, and increased synthesis of IL-1β, IL-6, IL-8, and IL-10 mRNA. A corresponding terminal pathway activation did not follow the C3 activation. These findings align with previous *in vitro* human whole blood studies, suggesting that C3-inhibition is relevant for further studies in the treatment of venous air embolism.

## Data Availability Statement

The raw data supporting the conclusions of this article will be made available by the authors upon request, without undue reservation.

## Ethics Statement

The animal study was reviewed and approved by The Norwegian Animal Research Authority (FOTS ID9466) and performed perthe Norwegian Laboratory Animal Regulations and EU directive2010/64/EU.

## Author Contributions

BS, TM, and EN conceptualized and designed the study. BS, EN, BN, and KD conducted animal experiments. BS, DC, HF, JL, KP, and AL acquired and analyzed the biological materials. BS and TB conducted the statistical analyses. BS, EN, and TM drafted the manuscript. All authors critically revised the manuscript and approved the final version.

## Funding

The study was funded by an unrestricted research grant from Northern Norway Regional Health Authority (Helse Nord RHF).

## Conflict of Interest

The authors declare that the research was conducted in the absence of any commercial or financial relationships that could be construed as a potential conflict of interest.

## Publisher’s Note

All claims expressed in this article are solely those of the authors and do not necessarily represent those of their affiliated organizations, or those of the publisher, the editors and the reviewers. Any product that may be evaluated in this article, or claim that may be made by its manufacturer, is not guaranteed or endorsed by the publisher.

## Abbreviations

CAU, complement activation units; CFT, clot formation time; IL, interleukin; IQR, interquartile range; NATEM, non-activated thromboelastometry test; NeF, nephritic factor; ROTEM, rotational thromboelastometry; TAT, thrombin-antithrombin complex; TCC, terminal complement complex; TNF, tumor necrosis factor.
